# The Role of Sintering Temperature and Dual Metal Substitutions (Al^3+^, Ti^4+^) in the Development of NASICON-Structured Electrolyte

**DOI:** 10.3390/ma14237342

**Published:** 2021-11-30

**Authors:** Hashlina Rusdi, Roshidah Rusdi, Shujahadeen B. Aziz, Abdullah Saad Alsubaie, Khaled H. Mahmoud, Mohd F. Z. Kadir

**Affiliations:** 1 Centre for Foundation Studies in Science, Universiti Malaya, Kuala Lumpur 50603, Malaysia; linaharun98@um.edu.my; 2 Centre for Nanomaterials Research, Institute of Science, Universiti Teknologi MARA, Shah Alam 40450, Malaysia; roshidahrusdi@yahoo.com; 3 Hameed Majid Advanced Polymeric Materials Research Lab., Physics Department, College of Science, University of Sulaimani, Sulaimani 46001, Iraq; shujahadeenaziz@gmail.com; 4 Department of Civil Engineering, College of Engineering, Komar University of Science and Technology, Sulaimani 46001, Iraq; 5 Department of Physics, Khurma University College, Taif University, Taif 21944, Saudi Arabia; asubaie@tu.edu.sa (A.S.A.); k.hussein@tu.edu.sa (K.H.M.)

**Keywords:** NASICON-structured, mechanical milling, glass ceramic electrolyte, impedance, dielectric properties, Li_1+x_Al_x_Ti_x_Sn_2−2x_P_3_O_12_

## Abstract

The aim of this study is to synthesize Li_1+x_Al_x_Ti_x_Sn_2−2x_(PO_4_) sodium super ion conductor (NASICON) -based ceramic solid electrolyte and to study the effect of dual metal substitution on the electrical and structural properties of the electrolyte. The performance of the electrolyte is analyzed based on the sintering temperature (550 to 950 °C) as well as the composition. The trend of XRD results reveals the presence of impurities in the sample, and from Rietveld Refinement, the purest sample is achieved at a sintering temperature of 950 °C and when x = 0.6. The electrolytes obey Vegard′s Law as the addition of Al^3+^ and Ti^4+^ provide linear relation with cell volume, which signifies a random distribution. The different composition has a different optimum sintering temperature at which the highest conductivity is achieved when the sample is sintered at 650 °C and x = 0.4. Field emission scanning electron microscope (FESEM) analysis showed that higher sintering temperature promotes the increment of grain boundaries and size. Based on energy dispersive X-ray spectroscopy (EDX) analysis, x = 0.4 produced the closest atomic percentage ratio to the theoretical value. Electrode polarization is found to be at maximum when x = 0.4, which is determined from dielectric analysis. The electrolytes follow non-Debye behavior as it shows a variety of relaxation times.

## 1. Introduction

Most researchers and engineers are rapidly changing their direction towards energy storage solutions, e.g., lithium-ion batteries and supercapacitors, due to growing awareness of the environmental impacts of fossil fuels and the resilience of energy grids worldwide. Lithium-ion batteries are used in various applications, such as automotive, aviation, electrical appliances, and smart devices [1,2]. Ceramic solid electrolyte (CSE) has many significant advantages, including high mechanical strength, excellent thermal stability together with electrochemical stability. These unique characteristics enable CSE to be helpful in machines that require highly durable materials [3,4]. CSE can eliminate several disadvantages, e.g., solvent evaporation, leakage, corrosion, and flammability [5].

Typically, CSE is a great Li-ion conductor compared to polymer and polymer/composite electrolytes, due to the presence of channels in which alkaline ions can migrate easily [6]. CSE also possesses better mechanical strength than polymer electrolyte. Some polycrystalline electrolytes that are classified under CSE are perovskite-type, which is a calcium titanium oxide mineral composed of calcium titanate (CaTiO_3_) [7]; garnet-type, such as Li_7_La_3_Zr_2_O_12_ [8]; sulfide-type, such as Li_3_PS_4_ and Li_4_SnS_4_ [9]; argyrodite-type, for example Li_6_PS_5_Br [10]; and NASICON-type, such as LiZr_2_(PO_4_)_3_ system [11]. Other types of CSE have amorphous structures instead of regular crystalline structures. Lithium phosphorus oxynitride (LiPON)-type—[12] and lithium thiophosphates (Li_2_S–P_2_S_5_) [13]—are some examples of amorphous CSE.

The performance of NASICON-type is strongly dependent on the materials and composition used in the framework. This is because the parent compound, LiM_2_(PO_4_)_3_ can be altered to various possible structures where M can be tin (Sn), titanium (Ti), germanium (Ge), hafnium (Hf) or zirconium (Zr) [14]. The PO4 tetrahedron and MO6 octahedron in NASICON structure acts as channels for alkali ions to move from one electrode to another [15]. Other researchers [16,17,18] reported that LiSn_2_P_3_O_12_ has great electrical and thermal stability and withstands shock and pressure. However, LiSn_2_P_3_O_12_ usually has a low conductivity value. This obstacle can be solved by substitution method using trivalent cation like gallium (Ga), indium (In), yttrium (Y), aluminum (Al), vanadium (V), iron (Fe), chromium (Cr) or scandium (Sc). In this work, dual metal element substitution method has been implemented using Al^3+^ and Ti^4+^. Hence the new formula after dual metal element substitution is Li_1+x_Al_x_Ti_x_Sn_2−2x_P_3_O_12_, where x is varied from 0.2 to 0.8. The substitution process creates positive charge deficiency, which is compensated by Li^−^ ion [19]. In this work the Li_1+x_Al_x_Ti_x_Sn_2−2x_(PO_4_)_3_sample will be produced using several sintering temperatures from 550 to 950 °C. The influence of sintering temperature and composition on the electrical and structural properties of the samples will be the main focus. Based on our knowledge, there is no report on Li_1+x_Al_x_Ti_x_Sn_2−2x_P_3_O_12_ electrolyte.

## 2. Experimental Procedures

### 2.1. Materials

Lithium oxide (Li_2_O) 99%, tin (IV) oxide (SnO_2_) 98%, ammonium dihydrogen phosphate (NH_4_H_2_PO_4_) 98%, titanium (IV) oxide (TiO_2_) 99.8% and aluminum oxide (Al_2_O_3_) 99.99%. These materials were procured from Sigma Aldrich (Saint Louis, MO, USA).

### 2.2. Li_1+x_Al_x_Ti_x_Sn_2−2x_P_3_O_12_ Preparation

Starting materials, e.g., SnO_2_, Li_2_O, TiO_2_, NH_4_H_2_PO_4_ and Al_2_O_3_ were ground and mixed using a planetary ball miller. The mixture was then placed in an alumina crucible and heated at 700 °C for 2 h. The pre-heating process was performed to eliminate H_2_O and NH_4_ from NH_4_H_2_PO_4_ to gain P_2_O_5_ [3]. The resultant mixtures were inserted into a jar filled with zirconium (Zr) balls (diameter = 0.4 cm). The milling process was conducted using a Fritsch 7 ball mill (Tencan, Changsha, China) at 500 rpm for 80 h. The height and inner diameter of the jar were 7.3 cm and 5.2 cm, respectively. The mixture was pressed at 7 tons of pressure using Specac Hydraulic Press to form an electrolyte pellet. The thickness of the electrolyte was from 1 to 13 mm. Different sintering temperatures such as 550, 650, 750, 850 and 950 °C were used for 8 h. A desiccator containing SiO_2_ gel was used to store the final electrolyte and to remove excess moisture.

### 2.3. Li_1+x_Al_x_Ti_x_Sn_2−2x_P_3_O_12_ Characterization

A Bruker AXS D8 Advance X-ray Diffraction spectrometer (Malvern Panalytical Ltd., Malvern, UK), (Cu-K radiation, 1.5406 Å) was employed to study the phase and structural properties of the electrolytein *2θ* range between 10 and 90°.The sample holder for XRD analysis was PW1813/26, 26 mm ∅ Steel Ring. The structure of Li_1+x_Al_x_Ti_x_Sn_2−2x_P_3_O_12_ was matched with the *R-3c* space group of LiSn_2_P_3_O_12_. XpertHighScore Plus software version 5.1 (Malvern Panalytical, Malvern, UK) was used to conduct structural studies by refinement method.Any changes on the surface of the electrolyte were analyzed using JEOL 7600F FESEM (Jeol Ltd., Tokyo, Japan). The electrolytes were examined under vacuum condition with accelerating voltage of 5 kV, and magnification of 20 kx. EDX was conducted using Oxford INCA X-Max 51-XMX 0021 (Oxford instruments nanoanalysis, Tokyo, Japan) integrated with FESEM (JEOL 7600F).

Solartron SI 1260 Impedance Analyzer (Artisan technology group, Champaign, IL, USA) was used to analyze the conductivity of each electrolyte in the frequency range of 1 Hz to 32 MHz. The value of conductivity (σ) from impedance spectra was obtained using the equation below:

(1) $$\sigma =\frac{d}{R_{bulk}A}$$

where *d* stands for the thickness of the electrolyte, *R_bulk_* is the bulk resistance and *A* is the interfacial contact area between the electrode and electrolyte. The dielectric constant may be used to portray the capacitive behavior of the electrolyte and to confirm the pattern of conductivity. The charge stored in the electrolyte is called the dielectric constant (*ε*′), while the energy dissipation is called the dielectric loss (*ε*″). Data from impedance analysis was used to obtain the dielectric parameters via the following equations [20]:

(2) $$\varepsilon^{\prime }=\frac{Z^{\prime \prime }}{\omega C_{o}{\left(Z^{\prime }\right)}^{2}+\omega C_{o}{\left(Z^{\prime \prime }\right)}^{2}}$$

(3) $$\varepsilon^{\prime \prime }=\frac{Z^{\prime }}{\omega C_{o}{\left(Z^{\prime }\right)}^{2}+\omega C_{o}{\left(Z^{\prime \prime }\right)}^{2}}$$

where *Z*′ and *Z*″ are the real (x-axis) and imaginary (y-axis) parts of the impedance. *C_o_* is the vacuum capacitance, and *ω* stands for angular frequency. The ionic-conducting behavior of a material may also be examined via electrical modulus analysis. The real and imaginary parts of modulus are *M*′ and *M*″, respectively, where *M*′ and *M*″ can be expressed as:

(4) $$M^{\prime }=\frac{{\left(\varepsilon^{\prime }\right)}^{2}}{{\left(\varepsilon^{\prime }\right)}^{2}+{\left(\varepsilon^{\prime \prime }\right)}^{2}}$$

(5) $$M\prime \prime =\frac{{\left(\varepsilon^{\prime \prime }\right)}^{2}}{{\left(\varepsilon^{\prime }\right)}^{2}+{\left(\varepsilon^{\prime \prime }\right)}^{2}}$$

From the peak in the plot of *M*″, relaxation time (*t_rex_*) was determined using the following equation:

(6) $$t_{rex}\omega_{peak}=1$$

where *ω_peak_* is the angular frequency of the relaxation peak.

## 3. Result and Discussion

### 3.1. X-ray Diffraction Study

**Figure 1:**
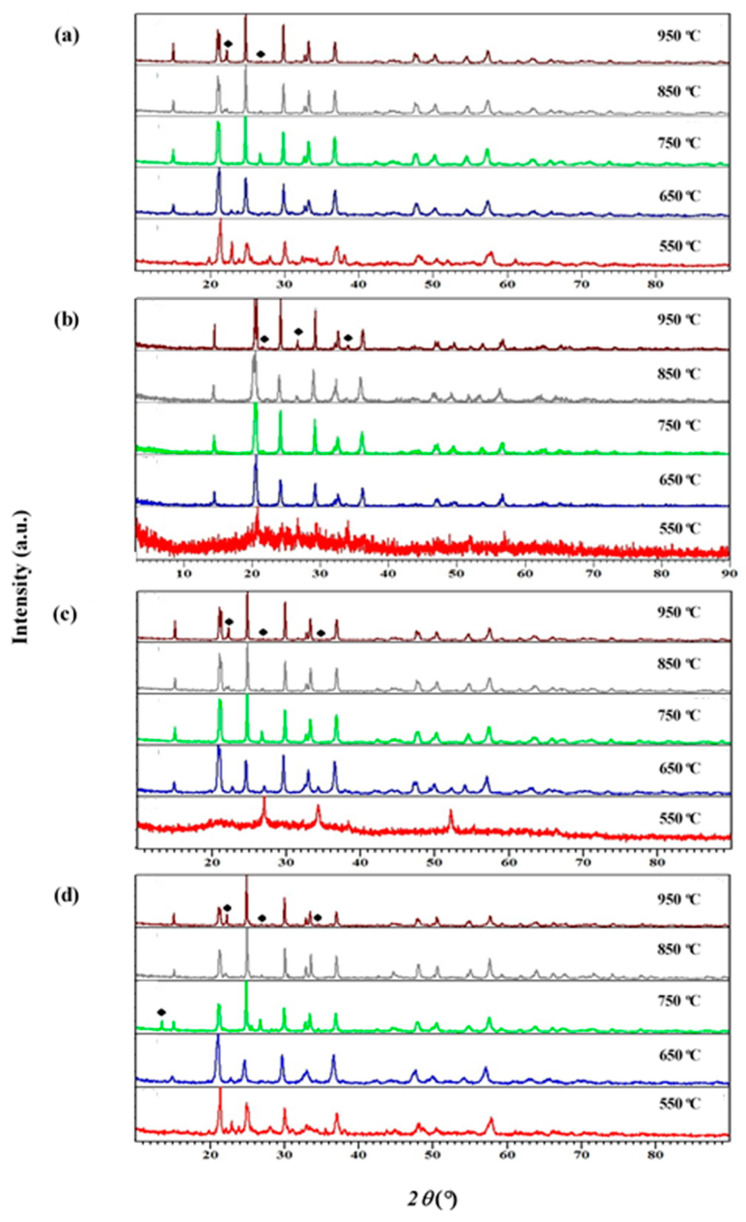
XRD diffractograms of Li_1+x_Al_x_Ti_x_Sn_2−x_P_3_O_12_ with x = (**a**) 0.2, (**b**) 0.4, (**c**) 0.6 and (**d**) 0.8 sintered at various sintering temperatures. The impurity peak of AlPO_3_ is marked as ♦.

Figure 1 shows the XRD patterns obtained for Li_1+x_Al_x_Ti_x_Sn_2−2x_(PO_4_)_3_ with x = 0.2, 0.4, 0.6 and 0.8 that are sintered at five different temperatures. XRD analysis is conducted to identify the formation of the compound either single phase or multi-phase as well as the existence of impurity in the compound. It is noticeable in Figure 1 that the samples sintered at 550 °C are more amorphous and when the samples are treated at higher temperature from 650 °C and above, the crystallinity of the sample increased. This is determined based on the sharpness and shape of the XRD peak. Diffraction peaks in all samples show the peaks are corresponding to LiSn_2_(PO_4_)_3_ and align with the International Center for Diffraction Data (ICDD) reference pattern (01-087-2078), which is reported in our previous work [21].

The samples sintered at 850 °C show almost pure samples with low impurity for all stoichiometries. However, the impurity peak of the AlPO_3_ phase is also observed in most samples. The impurity peaks are more obvious for the samples with x = 0.8. For the rest, the impurity peak to the signal ratio is quite low. It is attributed to a high amount of substituent, which causes Al^3+^ ions to react with PO^3−^ ions due to the strength of their charge interaction. Al^3+^ loses 3 electrons while PO^3−^ receives 3 electrons, thus producing high AlPO_3_ impurities [22]. This is also due to several factors such as lattice mismatch, ionic radius and lattice size [23]. These factors can be investigated using Rietveld Refinement method with the structural ICSD reference 83831 for LiSnPO_4_.

**Figure 2:**
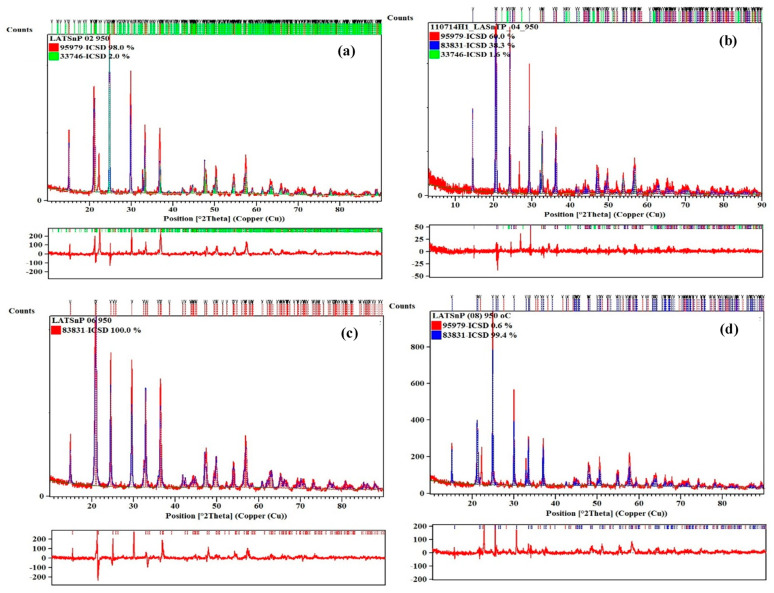
XRD Refinement results of Li_1+x_Al_x_Ti_x_Sn_2−2x_(PO_4_)_3_ when x = (**a**) 0.2, (**b**) 0.4, (**c**) 0.6 and (**d**) 0.8 sintered at 950 °C.

The refinements are done for samples from 650 °C and above due to the crystallinity issues. All samples that are sintered at 550 °C portray amorphous structures. From Rietveld Refinement analysis, all samples have the lowest impurity amount at a sintering temperature of 950 °C; thus, Figure 2 shows the XRD Refinement results in Li_1+x_Al_x_Ti_x_Sn_2−2x_(PO_4_)_3_ sintered at 950 °C. All parameters obtained from refinement analysis are tabulated in Table 1. The impurity presence in the compound is mostly AlPO_4_, while some are TiO_2_ and SnO_2_. There is no trend of impurity amount with the increased amount of substituent. The purest compound with impurity-free is when x = 0.6 is sintered at 950 °C. When more substituents are added into the system, the cell parameter and the cell volume become smaller than LSP. The refinement results show that the problem with this system or compound is the element vacancies in the system.

**Figure 3:**
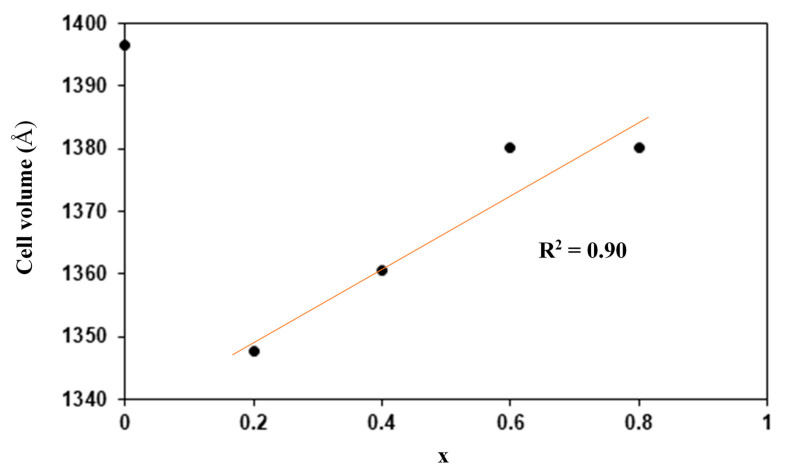
Cell volume versus x values.

Table 1 reveals that aluminum and titanium substitution affect the lattice parameters of the LSP. When aluminum and titanium content increases, the cell volume decreases when x = 0 to x = 0.2 and increased back when more aluminum and titanium are substituted as shown in Figure 3. This is due to the smaller ionic radius of Al^3+^ (0.57 Å) and larger Ti^4+^ (0.75 Å) compared to Sn^4+^ (0.65 Å). Linear relation can be seen between the cell volume and x. This phenomenon indicates that these samples obey Vegard’s Law [24]. Kahlaoui et al. [25] reported that due to the linearity of cell volume–composition relations, the distribution of Li and Ba in Ba_x/2_Li_1-x_Ti_2_(PO_4_)_3_ NASICON-based electrolyte is expected to be random. Thus, Al^3+^ and Ti^4+^ in Li_1+x_Al_x_Ti_x_Sn_2−2x_(PO_4_)_3_ system are in random distribution.

### 3.2. Conductivity and ImpedanceStudy

Conductivity measurement is done on all samples and sintering temperatures. The conductivity values are summarized in Table 2. The different sample has different optimum temperatures. We can observe that the highest conductivity for x = 0.2 and 0.4 is at a sintering temperature of 650 °C, while that for x = 0.6 and 0.8 is at 550 °C and 850 °C respectively. The result shows that lower sintering temperature is more suitable for glass-ceramic to give relatively good conductivity values and applied as solid electrolyte. Using low sintering temperatures, the inter-atom and intermolecular forces are not strong, making the process of electron or ion conduction easy, and flowing to complete the circuit in the system. Narayanan et al. [26] reported the same phenomenon wherein high sintering temperatures reduce ionic conductivity. The authors also stated that the synthesis conditions and sintering temperature greatly influence the conductivity of the NASICON samples.

**Figure 4:**
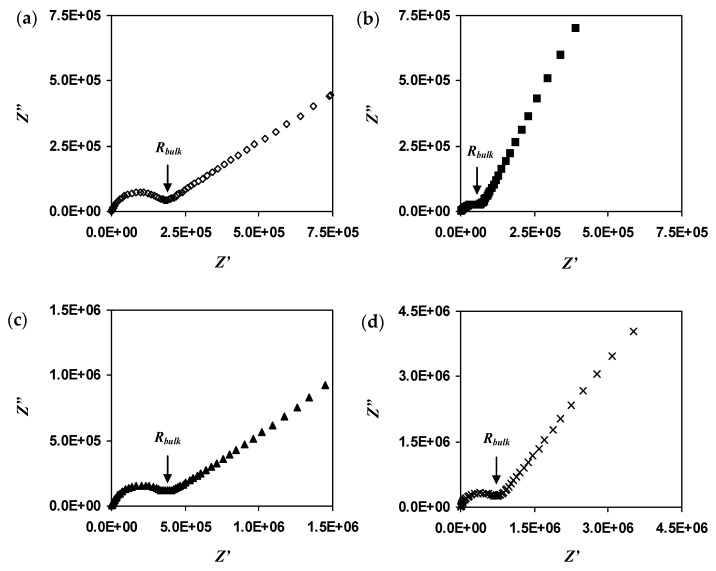
Nyquist plot of the Li_1+x_Al_x_Ti_x_Sn_2-2x_P_3_O_12_with x equal to (**a**) 0.2, (**b**) 0.4, (**c**) 0.6 and (**d**) 0.8 sintered at 650 °C.

As the Li_1+x_Al_x_Ti_x_Sn_2−2x_(PO_4_)_3_ sample has the highest conductivity at sintering temperature of 650 °C, Nyquist plots of all samples at sintering temperature of 650 °C are chosen for a better comparison purpose. Figure 4 shows the Nyquist plot of Li_1+x_Al_x_Ti_x_Sn_2−2x_(PO_4_)_3_ where x = 0.2, 0.4, 0.6, 0.8 and is sintered at 650 °C. The value of *R_bulk_* for this kind of plot is taken from the meeting point of the titled line and the semicircle. The semicircle at higher frequency region is due to the conduction of ions in the bulk of the electrolyte while the tilted line at low frequency indicates polarization effects [27]. It is obvious that the bulk resistance of the electrolyte is reduced and is the smallest when x = 0.4. As the amount of substituent increases, the bulk resistance is observed to increase. The highest conductivity achieved is 4.74 × 10^−6^ S cm^−1^ when x = 0.4 (Table 2). Rao et al. [28] stated that the differences in conductivity are typically related to the connectivity between grains, which have a higher concentration of imperfections near the grain boundary. The authors reported a similar trend of conductivity variation where the highest conductivity value is 4.25 × 10^−6^ S cm^−1^ for LiTi_2_(PO_4_)_3_ system. The inclusion of x = 0.4 of Al^3+^ has optimized the conductivity to 2.5 × 10^−6^ S cm^−1^ for Li_1+x_Al_x_Sn_1.2+x_P_3_O_12_-based solid electrolyte, which is reported by Lu et al. [29]. From Table 1, the conductivity values change more on the *c* side and the cell volume increases as x value increases. When the cell volume increases, this may cause the ion to move more easily in the cell, which in turn enhances the conductivity values. However, too large a volume can lead to conductivity decrement as ions require more energy to move to neighboring sites.

### 3.3. FESEM Analysis

**Figure 5:**
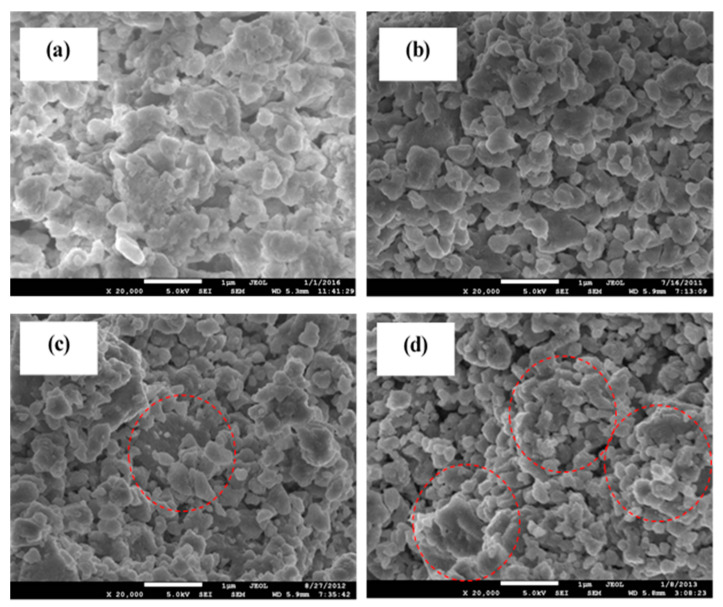
FESEM micrograph of the Li_1+x_Al_x_Ti_x_Sn_2-2x_P_3_O_12_ with x equal to (**a**) 0.2, (**b**) 0.4, (**c**) 0.6 and (**d**) 0.8 sintered at 650 °C. Dotted red circle represents the bimodal grain size distribution.

FESEM micrographs of samples sintered at 650 °C are selected as the highest conductivity is obtained at this sintering temperature, as can be seen in Figure 5. FESEM micrographs of all compositions show an irregularity in shape, and some have a flaky type of morphology along high agglomeration. When x = 0.2, the surface has some large grain structure with size more than 1 µm, while most grain structure is less than 1 µm in x = 0.4. FESEM micrographs of x = 0.6 and 0.8 possess a bimodal grain size distribution with small grains localized around larger grains. Narváez-Semanate et al. [30] reported that the sample with bimodal grain size distribution in the Li_1+x_Al_x_Ti_2−x_(PO_4_)_3_ system has low ionic conductivity value. Furthermore, among all compositions, the sample with x = 0.4 has the most consistent particle arrangement and size distribution, while x = 0.2, 0.6 and 0.8 has larger grain structures. The pathway of ions and electrons to conduct is easier in compounds with consistent particle size distribution [31].Results of FESEM analysis are in good agreement with the conductivity results in Table 2.

**Figure 6:**
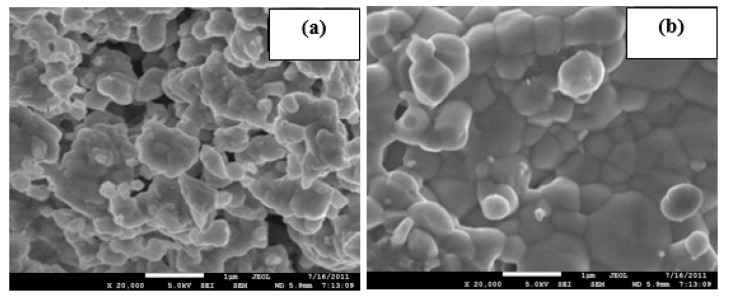
FESEM micrograph of the Li_1.4_Al_0.4_Ti_0.4_Sn_1.2_P_3_O_12_ (x =0.4) sintered at (**a**) 750 °C and (**b**) 950 °C.

**Figure 7:**
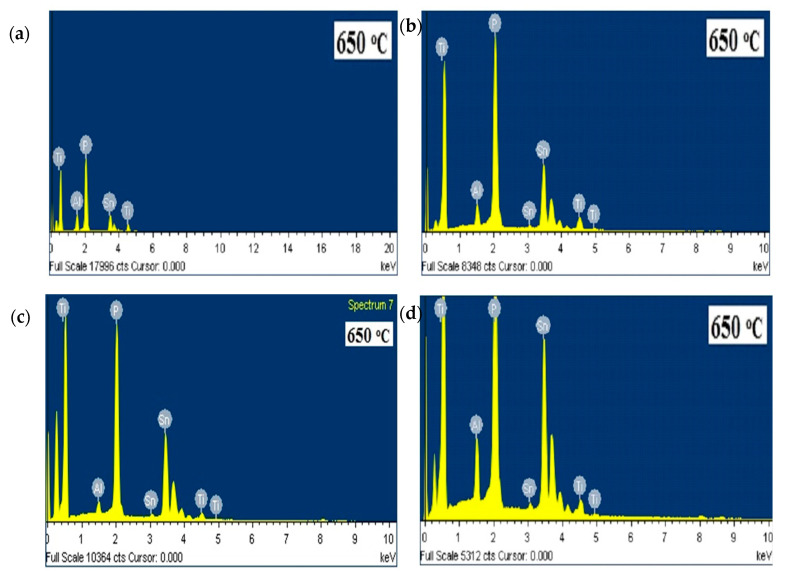
EDX spectrum ofLi_1+x_Al_x_Ti_x_Sn_2-2x_P_3_O_12_ with (**a**) x = 0.2, (**b**) x = 0.4, (**c**) x = 0.6 and (**d**) x = 0.8 sintered at 650 °C.

Figure 6 illustrates the FESEM micrographs of the Li_1.4_Al_0.4_Ti_0.4_Sn_1.2_P_3_O_12_ sintered at low and high temperatures. Electrolyte sintered at 750 °C shows an increase in grain size, while the grain size is further increased for 950 °C and clear grain boundaries can be seen. The increment in grain size is usually due either to re-crystallization or the existing defects in the crystal [32,33]. These results are in good agreement with XRD analysis, in which most samples experience increments in crystallinity at higher sintering temperature, as shown in Figure 1. Liu et al. [34] reported the same pattern of grain size growth for the Li_1.3_Al_0.3_Ti_1.7_(PO_4_)_3_ system. The authors also stated that sintering temperature will affect the grain size and the resistance of the electrolyte. This explains the pattern of the conductivity value in Table 2 where most samples possess low conductivity when sintered at high temperature. Figure 7 shows the EDX plot, and the average value of atomic percentages of elements is tabulated in Table 3. Based on EDX analysis, x = 0.4 produced the closest atomic percentage ratio to the theoretical value.

### 3.4. Dielectric Analysis

**Figure 8:**
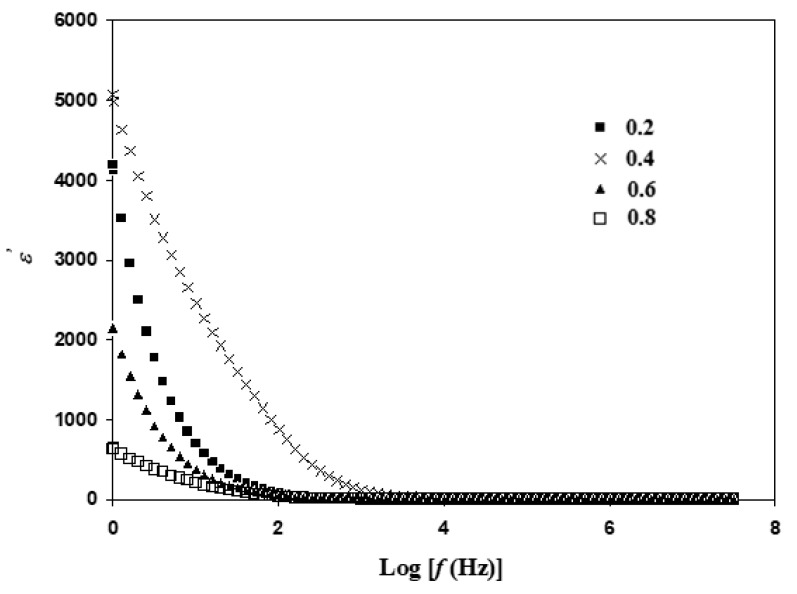
Variation of ε′ at different frequencies for Li_1+x_Al_x_Ti_x_Sn_2−2x_(PO_4_)_3_ at sintering temperature of 650 °C.

**Figure 9:**
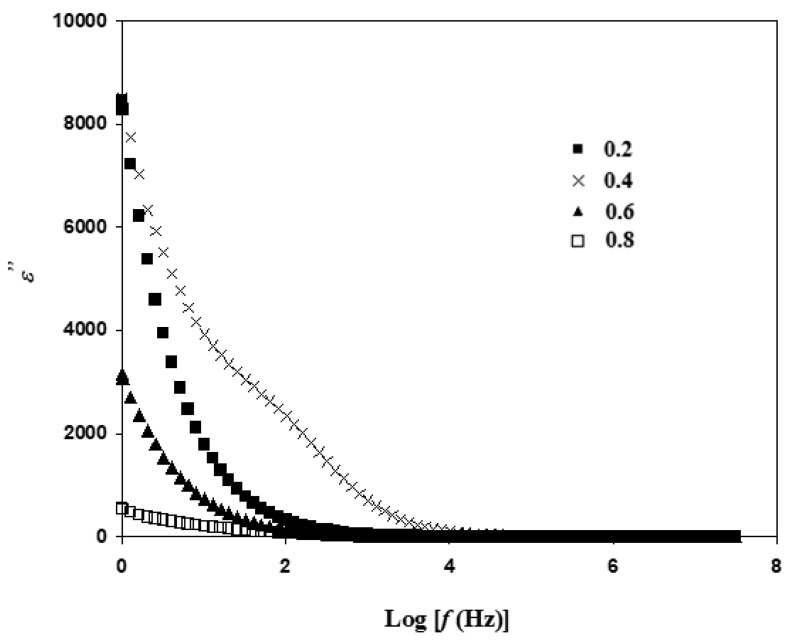
Variation of $\varepsilon^{\prime \prime }$ at different frequencies for Li_1+x_Al_x_Ti_x_Sn_2−2x_(PO_4_)_3_ at sintering temperature of 650 °C.

Highest conductivity of Li_1+x_Al_x_Ti_x_Sn_2−2x_P_3_O_12_ is obtained at sintering temperature of 650 °C; thus, dielectric study at this sintering temperature is chosen to verify the pattern of conductivity. Dielectric analysis is a crucial method for identifying ionic transport and phase transition mechanism in a system. The pattern of ε′ is displayed in Figure 8, which has almost the same trend as$\;\varepsilon^{\prime \prime }\;$in Figure 9, where it is high at low frequency region [35].

As observed in Figure 8, the value of ε′ is low at high frequency region (Log *f* > 3) while high at low frequency region [36]. Rao et al. [28] reported that polarization happens at low frequency as ions can form proper charge double layers at the surface of the electrode. ε′ is observed to approach zero as Log *f* is more than 3. At rapid rate of electric field, charge carriers experience unstable flow, including collisions among charge carries, which disable proper formation of charge double-layer [37]. The value of ε′ is at maximum when x = 0.4. Thus, it is proven that in Li_1+x_Al_x_Ti_x_Sn_2−2x_(PO_4_)_3_, the number of charge carriers is the largest when x = 0.4. The trend of conductivity in Table 2 (at sintering temperature of 650 °C) is further verified, as it is consistent with the trend of ε′ in Figure 8.

The loss of energy of Li_1+x_Al_x_Ti_x_Sn_2−2x_(PO_4_)_3_at 650 °C is shown in Figure 9. The value of $\varepsilon^{\prime \prime }\;$for a sample with x = 0.4 is at maximum, and drops as x value changes to 0.2, 0.6 and 0.8. This shows that Li_1.4_Al_0.4_Ti_0.4_Sn_1.2_(PO_4_)_3_ has more free ions or charge carriers compared to other compositions. More energy loss is observed as more ionic collision occurs. The pattern of dielectric constant and loss in this study is similar to other NASICON-based solid electrolyte works [28,38]. These authors stated that their NASICON-based electrolytes have the behavior of an ionic conductor. Meena et al. [39] reported that the hump in dielectric of Co_3−x_Mn_x_O_4_ ceramic is due to the presence of dielectric anomaly peak. According to the work by Hyatt et al. [40] and Luo et al. [41], the hump in the real part of permittivity is due to the presence of second phase and impurity.

### 3.5. Electric Modulus Atable Analysis

**Figure 10:**
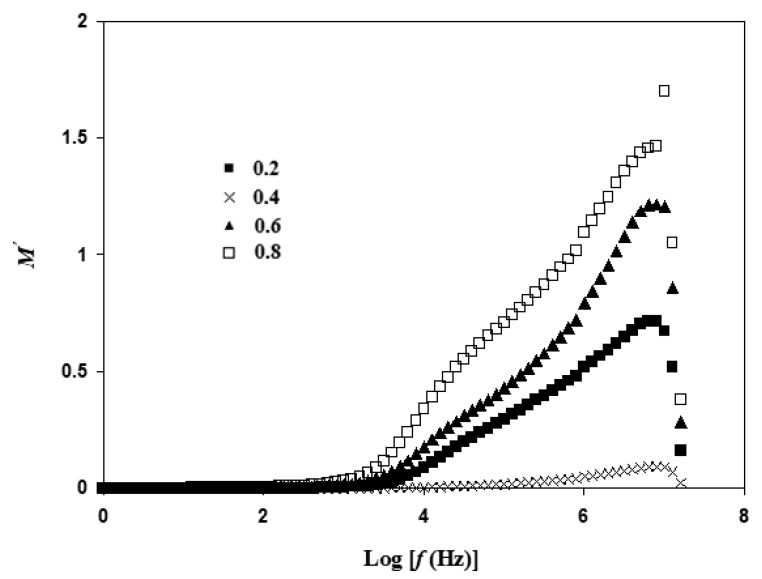
Variation of *M*′ at different frequencies for Li_1+x_Al_x_Ti_x_Sn_2−2x_(PO_4_)_3_ at sintering temperature of 650 °C.

The electrical properties of Li_1+x_Al_x_Ti_x_Sn_2−2x_(PO_4_)_3_ is further studied using electrical modulus, which analyzes the response of Li^+^ ions in the presence of electric field. Modulus is used to examine ionic conductivities in correlation with the ionic process and conductivity relaxation. It can be observed that the pattern of *M*′ in Figure 10 possesses a peak at ~Log *f* = 7 for all compositions. The presence of this peak is common in a conductor of ion.

Relaxation process is usually located at the high frequency region, while conduction process is at low frequency region [42]. The value of *M*′ is low and almost approaching zero from Log *f* = 0 to Log *f* = 3. Tripathi et al. [43] reported that at low frequency region, electrode polarization is dominant. This outcome tallies with the results of conductivity in Table 2.

**Figure 11:**
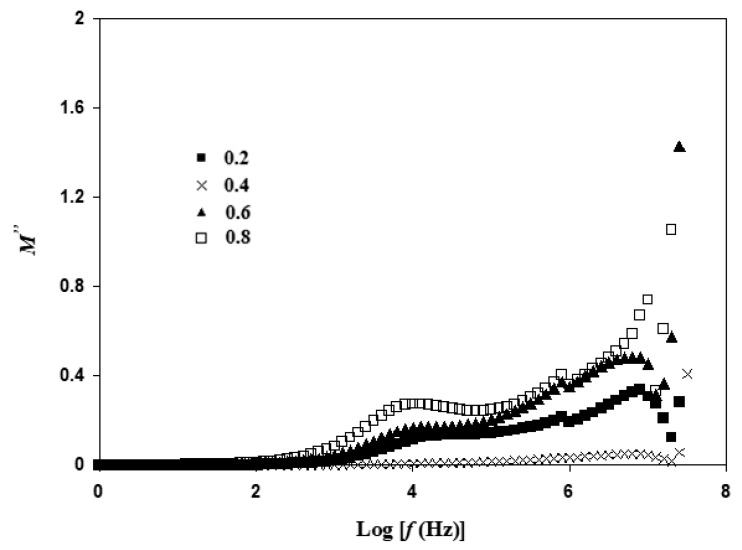
Variation of *M*″ at different frequencies for Li_1+x_Al_x_Ti_x_Sn_2−2x_(PO_4_)_3_ at sintering temperature of 650 °C.

Figure 11 illustrates the effect of frequency on *M*″. The peak of *M*″ shifted towards higher frequency from x = 0.2 to x = 0.4 and shifted back towards lower frequency regions for x = 0.6 and 0.8. Nikam and Deshpande [44] stated that variation of relaxation of charge carriers signifies that the system follows non-Debye-type behavior. This outcome is similar to the NASICON-type structure reported by Arumugam et al. [45] with the Li_1.3_Al_0.3_Ti_1.7_(PO_4_) system.

The *t_rex_* of each electrolyte is tabulated in Table 4. *t_rex_* is 98 × 10^−8^ s for x = 0.2 and reduces to 1.57 × 10^−8^ s when x = 0.4. The trend of *t_rex_* supports the trend of conductivity. The presence of relaxation is attributed to conduction of free ions in alternating electric fields. It is noticeable that there are several humps in the frequency range between Log *f* = 4 to 6 in all composition. This is due to the presence of grain and grain boundaries. Polarization of ceramic is highly influenced by the existence of grain boundaries [46]. Supriya et al. [47] stated that a high number of grain boundaries produce more dipole formation.

## 4. Conclusions

The main objective of this study has been achieved as different sintering temperature yields different grain size, grain boundary visibility, conductivity and impurity content for NASICON electrolyte with the Li_1+x_Al_x_Ti_x_Sn_2−2x_(PO_4_)_3_ system. Li_1+x_Al_x_Ti_x_Sn_2−2x_(PO_4_)_3_ systems have been successfully prepared using mechanical milling method at 550, 650, 750, 850 and 950 °C. Enhancement of crystallinity with increasing sintering temperature can be seen in XRD analysis. The purest Li_1+x_Al_x_Ti_x_Sn_2−2x_(PO_4_)_3_ system can be obtained at sintering temperature of 950 °C and x = 0.6. Thus, in applications that require pure samples, this sintering temperature and composition can be used. The Li_1+x_Al_x_Ti_x_Sn_2−2x_(PO_4_)_3_ system obeys Vegard’s Law: a linear pattern of cell volume can be observed as Al^3+^ and Ti^4+^ are added. The optimum sintering temperature to obtain highest conductivity for samples with x = 0.2, 0.4, 0.6 and 0.8 are 650 °C, 650 °C, 550 °C and 850 °C, respectively. Thus, it can be verified that sintering temperature has a great influence on conductivity. The Li_1+x_Al_x_Ti_x_Sn_2−2x_(PO_4_)_3_ system has the highest conductivity at sintering temperature of 650 °C and x = 0.4. Growth in grain size and grain boundary can be seen at high sintering temperature. Maximum electrode polarization and dielectric constant are obtained when x = 0.4. The Li_1+x_Al_x_Ti_x_Sn_2−2x_(PO_4_)_3_ system follows non-Debye behavior as it shows a variation of relaxation times. Thus, it can be concluded that different sintering temperatures and compositions produced Li_1+x_Al_x_Ti_x_Sn_2−2x_(PO_4_)_3_ with various properties (e.g., purity, structure, crystallinity, conductivity and dielectric). Hence, the selection of sintering temperature as well as composition should be aligned with the desired application. The performance of the electrolyte can be further enhanced in order to be useful in energy devices. This ceramic electrolyte can be used in hybrid polymer–ceramic electrolytes where polymers like polyethylene oxides (PEO) and polyvinylidene fluoride (PVDF) are used. This can improve electrolyte flexibility and provide more channel for ions to be conducted. Thus, a number of improvements can be made in the future. Other than that, different synthesis approaches (e.g., sintering temperature, size of balls in the ball miller, sintering time, composition, compatibility of materials and ambience for the analysis) might yield a different result.

## Figures and Tables

**Table 1:** Lattice parameters and side occupancy factor (s.o.f.) of Li_1+x_Al_x_Ti_x_Sn_2−x_P_3_O_12_ (x = 0.2, 0.4, 0.6, 0.8) sintered at 950 °C.

Sample	Impurity (%)	a(=b)/Å	c (Å)	V (Å^3^)	c/a	R_w_	χ^2^	s.o.f of Li in 6b	s.o.f of Sn in 12c	s.o.f of Al in 12c	s.o.f of P in 3b	s.o.f of O
LSP (83831)	-	8.63	21.53	1389.82	2.49	-	-	1.00	1.00	0.00	1.00	1.00
x = 0	19.0	8.63	21.66	1396.48	2.51	9.60	3.27	1.00	1.00	-	1.00	1.00
x = 0.2	2.0	8.55	21.29	1347.56	2.49	22.06	3.69	1.00	0.79	0.09	0.11	0.70
x = 0.4	1.6	8.58	21.33	1360.55	2.49	40.72	1.24	1.00	0.45	0.50	0.05	0.70
x = 0.6	-	8.61	21.50	1380.20	2.50	16.89	2.45	1.00	0.88	0.30	0.30	1.00
x = 0.8	0.6	8.61	21.50	1380.20	2.50	20.68	2.45	1.00	0.39	0.35	0.13	1.00

**Table 2:** Conductivity values for all samples at different sintering temperatures.

Sintering Temperature (°C)	Conductivity (S cm^−1^)			
	x = 0.2	x = 0.4	x = 0.6	x = 0.8
550	4.03 × 10^−6^	2.13 × 10^−6^	2.83 × 10^−6^	3.59 × 10^−7^
650	4.08 × 10^−6^	4.74 × 10^−6^	1.41 × 10^−6^	1.41 × 10^−7^
750	1.65 × 10^−6^	1.60 × 10^−6^	1.87 × 10^−6^	9.25 × 10^−8^
850	1.03 × 10^−6^	7.12 × 10^−7^	2.65 × 10^−6^	3.57 × 10^−6^
950	2.46 × 10^−6^	3.85 × 10^−6^	2.82 × 10^−6^	2.62 × 10^−6^

**Table 3:** Average value of atomic percentages of elements for all electrolytes at 650 °C.

x	Elements	Atomic Percentage (%)	
		Theory	Calculated
0.2	Sn	32	16.1
	P	60	63.0
	Al	4	9.3
	Ti	4	11.6
0.4	Sn	24	36.7
	P	60	53.0
	Al	8	3.5
	Ti	8	6.8
0.6	Sn	16	30.8
	P	60	61.9
	Al	12	3.7
	Ti	12	3.6
0.8	Sn	8	26.8
	P	60	60.9
	Al	16	8.4
	Ti	16	3.9

**Table 4:** Relaxation time for Li_1+x_Al_x_Ti_x_Sn_2−2x_(PO_4_)_3_ (x = 0.2, 0.4, 0.6, 0.8).

x	*t_rex_* (s)
0.2	1.98 × 10^−8^
0.4	1.57 × 10^−8^
0.6	2.49 × 10^−8^
0.8	1.57 × 10^−5^

## Data Availability

Not applicable.
